# Hepatitis C Virus Driven AXL Expression Suppresses the Hepatic Type I Interferon Response

**DOI:** 10.1371/journal.pone.0136227

**Published:** 2015-08-27

**Authors:** Scott A. Read, Enoch S. Tay, Mahsa Shahidi, Kate S. O’Connor, David R. Booth, Jacob George, Mark W. Douglas

**Affiliations:** 1 Storr Liver Centre, Westmead Millennium Institute, University of Sydney at Westmead Hospital, Westmead, Australia; 2 Centre for Immunology and Allergy Research, University of Sydney at Westmead Hospital, Westmead, Australia; 3 Centre for Infectious Diseases and Microbiology, Marie Bashir Institute for Infectious Diseases and Biosecurity, University of Sydney at Westmead Hospital, Westmead, Australia; University of Montreal Hospital Research Center (CRCHUM), CANADA

## Abstract

Treatment of chronic hepatitis C virus (HCV) infection is evolving rapidly with the development of novel direct acting antivirals (DAAs), however viral clearance remains intimately linked to the hepatic innate immune system. Patients demonstrating a high baseline activation of interferon stimulated genes (ISGs), termed interferon refractoriness, are less likely to mount a strong antiviral response and achieve viral clearance when placed on treatment. As a result, suppressor of cytokine signalling (SOCS) 3 and other regulators of the IFN response have been identified as key candidates for the IFN refractory phenotype due to their regulatory role on the IFN response. AXL is a receptor tyrosine kinase that has been identified as a key regulator of interferon (IFN) signalling in myeloid cells of the immune system, but has not been examined in the context of chronic HCV infection. Here, we show that AXL is up-regulated following HCV infection, both *in vitro* and *in vivo* and is likely induced by type I/III IFNs and inflammatory signalling pathways. AXL inhibited type IFNα mediated ISG expression resulting in a decrease in its antiviral efficacy against HCV *in vitro*. Furthermore, patients possessing the favourable *IFNL3* rs12979860 genotype associated with treatment response, showed lower *AXL* expression in the liver and a stronger induction of *AXL* in the blood, following their first dose of IFN. Together, these data suggest that elevated AXL expression in the liver may mediate an IFN-refractory phenotype characteristic of patients possessing the unfavourable rs12979860 genotype, which is associated with lower rates of viral clearance.

## Introduction

Disruption of the innate immune response has long been considered the basis for establishment of chronic hepatitis C virus (HCV) infection [1]. More recently, “interferon refractoriness” has been described in the HCV infected liver, characterized by high baseline interferon stimulated gene (ISG) expression, and limited response to exogenous interferon (IFN) [2,3]. Negative regulators of the IFN signalling pathway have been obvious targets in the search for a mechanism behind these observations, including the suppressors of cytokine signalling (SOCS) proteins, protein inhibitor of activated STAT (PIAS) and ubiquitin specific peptidase 18 (USP18); all of which are up-regulated by HCV [4–6].

While the exact mechanism underlying the development of interferon refractoriness remains uncertain, simultaneous genome-wide association studies (GWASs) identified a cluster of single nucleotide polymorphisms (SNPs) near the *IFNL3* gene that predict response to IFNα and RBV treatment in genotype 1 patients [7–9]. Patients possessing the “non-responder” SNP also demonstrated the IFN refractory phenotype, suggesting a causal link. While the functional relevance of the *IFNL3* SNPs remain uncertain, patients possessing the favourable haplotype may produce more IFNL3 [7,8], or may *not* produce a novel intrahepatic IFN termed IFNL4, which maintains low ISG expression, and facilitates viral clearance by maintaining sensitivity to IFN stimulation [10].

AXL is a member of the TAM family of receptor tyrosine kinases, and acts as a negative regulator of innate immune and inflammatory signalling, primarily in myeloid cells of the immune system [11,12]. The regulatory role of AXL in epithelial tissue is less well understood, and is of particular relevance in the HCV infected liver due to its regulation of SOCS3. Following stimulation of mouse dendritic cells with IFNα, AXL was shown to highjack IFNα signalling by binding to the IFNα receptor IFNAR1 to prevent its signal transduction. Simultaneously, AXL mediated the formation of STAT1 homodimers (rather than the ISG stimulating STAT1:STAT2 heterodimers), to induce the expression of SOCS1 and SOCS3 [12].

We have previously shown that AXL is induced by HCV infection *in vitro* [13] and have subsequently chosen to examine the functional relevance of AXL up-regulation by HCV. Here we confirm that AXL is up-regulated during HCV infection *in vitro* and *in vivo* and that AXL expression in the liver is driven primarily by type I/III IFN signalling, as well as inflammatory signalling pathways. Moreover, AXL reduces activation of the innate immune response by IFNα in hepatocytes, limiting the antiviral response to HCV. Lastly, patients possessing the theIFNL3 rs12979860 responder SNP (CC) demonstrated reduced baseline AXL expression in the liver and a stronger peripheral blood mononuclear cell (PBMC) AXL up-regulation after the first injection of IFN.

## Material and Methods

### Patient samples

Liver biopsies were collected from untreated patients chronically infected with HBV (n = 23), patients with HCV genotype 1/3 infection and low fibrosis (n = 31/n = 24) and HCV genotype 1 infection with high fibrosis (n = 16, Metavir score 3–4). All ‘low fibrosis’ samples were confirmed histologically to have Metavir fibrosis score ≤1 and steatosis Grade ≤ 1, unless otherwise stated. Peripheral blood mononuclear cell (PBMC) RNA was obtained from 15 healthy controls, as well as 18 genotype 1 HCV patients at baseline and 12 h after the first interferon injection using PAXgene blood RNA tubes (Qiagen). All genotype 1 patients were genotyped for the rs12979860 IFNL3 SNP by Taqman genotyping as described in [14]. Ethics approval and patient consent for the research use of blood and biopsies was provided for all samples.

### Cell culture and virus infection

Huh-7 and HepG2 (human hepatoma) cell lines were grown in Dulbecco’s minimal essential medium (DMEM) with 10% fetal bovine serum (both kindly provided by Dr John Dr John McLauchlan, MRC-University of Glasgow Centre for Virus Research, UK). HCV RNA was transcribed, electroporated, and baculovirus transfection of HepG2 cells was performed as in [15]. Fugene HD (Promega) was used to transfect full length HCV (JFH1 strain) and subgenomic replicon (SGR) RNA into Huh-7 cells to examine short term (24 h) viral RNA replication.

### Cytokines

IFNα was obtained from Roche (Roferon-A), IFNβ from Biogen Idec (Avonex), IFNγ from Jomar Bioscience and recombinant IFNλ3 andIL6 were obtained from R&D Systems.

### Chemical inhibition, gene knockdown and overexpression

Huh-7 cells were treated for 24 h with 50 μM SP600125 or 25 μM BAY11-7082, to inhibit JNK or NFκB signalling respectively. To inhibit STAT mediated signalling, Huh-7 cells were transfected with 10 nM siRNA against STAT1 (Sigma Aldrich EHU071921) or STAT3 (Sigma Aldrich EHU122051) for 24 h, with RNAiMax (Life Technologies) or AllStars scrambled siRNA (Qiagen) as a control. AXL expression was knocked down using 25 μM Mission siRNA (Sigma Aldrich EHU081461) for 24 h, prior to 24 h of IFN treatment. A stable polyclonal Huh-7 cell line overexpressing AXL was established by transfecting the pCMV6-AXL ORF plasmid (Origene) with Fugene HD (Promega) according to the manufacturers protocol, and selecting with G418 (Life Technologies). The empty vector pCMV6-Entry was maintained under G418 selection as well, and was used as a control.

### GAS, ISRE, AP-1 and NF-κB activity reporter assays

Firefly luciferase reporter plasmids containing the gamma-activated sequence (GAS), interferon-stimulated response element (ISRE), activator protein 1 (AP-1) and nuclear factor kappa-light-chain-enhancer of activated B cells (NFκB) were transiently transfected into Huh-7 using Fugene HD (Promega) as previously described [13]. IFNα was added 48 h post-electroporation and luciferase activity was quantified using the VICTOR plate reader and normalized to total protein content.

### Real-time PCR

cDNA was synthesised from 500 ng of RNA using Promega the MMLV Reverse Transcriptase Kit according to the manufacturer’s protocol. qPCR was performed on the Rotor-Gene 3000 or 6000 platform (Qiagen) using either Taqman or SYBR green protocols to amplify the HCV 5’UTR (Applied Biosystems Pa03453408), AXL (AGCGATGTGTGGTCCTTCG, TCCCTGGCGCAGATAGTCAT), SOCS3 (CACATGGCACAAGCACAAGA, CCCTCCAACACATTCCAGGT), ISG15 (CGCAGATCACCCAGAAGATC, GCCCTTGTTATTCCTCACCA), USP18 (CAGACCCTGACAATCCACCT, AGCTCATACTGCCCTCCAGA) and Viperin (CTTTTGCTGGGAAGCTCTTG, CAGCTGCTGCTTTCTCCTCT). All samples were normalized to 18s (Applied Biosystems 4319413E).

### Western blotting

Protein lysates were electrophoretically run on a 10% sodium dodecyl sulphate poly-acrylamide gel (SDS-PAGE) and transferred at 80 V for 1.5 h onto a nitrocellulose membrane. Membranes were blotted using AXL and pAXL (RnD Systems AF154, AF2228), Myc (Cell Signaling 2272), STAT1 and p-STAT1 (Santa Cruz Biotechnology SC-345, Cell Signalling 9167) and β-actin (Sigma-Aldrich A1978) antibodies, followed by peroxidise-conjugated secondary antibodies. Probed membranes were visualized using the Supersignal West Pico chemiluminescence kit (Pierce Endogen) and exposed to X-ray film.

### Identification of transcription factor binding sites

To identify transcription factor binding sites within the AXL promoter/gene, the UCSC genome browser (http://genome.ucsc.edu) was interrogated for experimentally validated chromatin immunoprecipitation (ChIP) data [16]. Evolutionarily conserved promoter/enhancer regions within the AXL gene were identified with ECR browser (http://ecrbrowser.dcode.org/) and transcription factor binding sites identified with rVista 2.0 (http://rvista.dcode.org/) [17,18].

### Data analysis

Quantitative data was expressed as mean ± standard error of the mean (SEM). Statistical analysis was performed using Graphpad Prism, comparing control to treated groups. Student’s t-tests were performed to compare individual treatments.

## Results

### AXL expression is induced by HCV infection *in vitro* and *in vivo* and is genotype dependent

**Fig 1:**
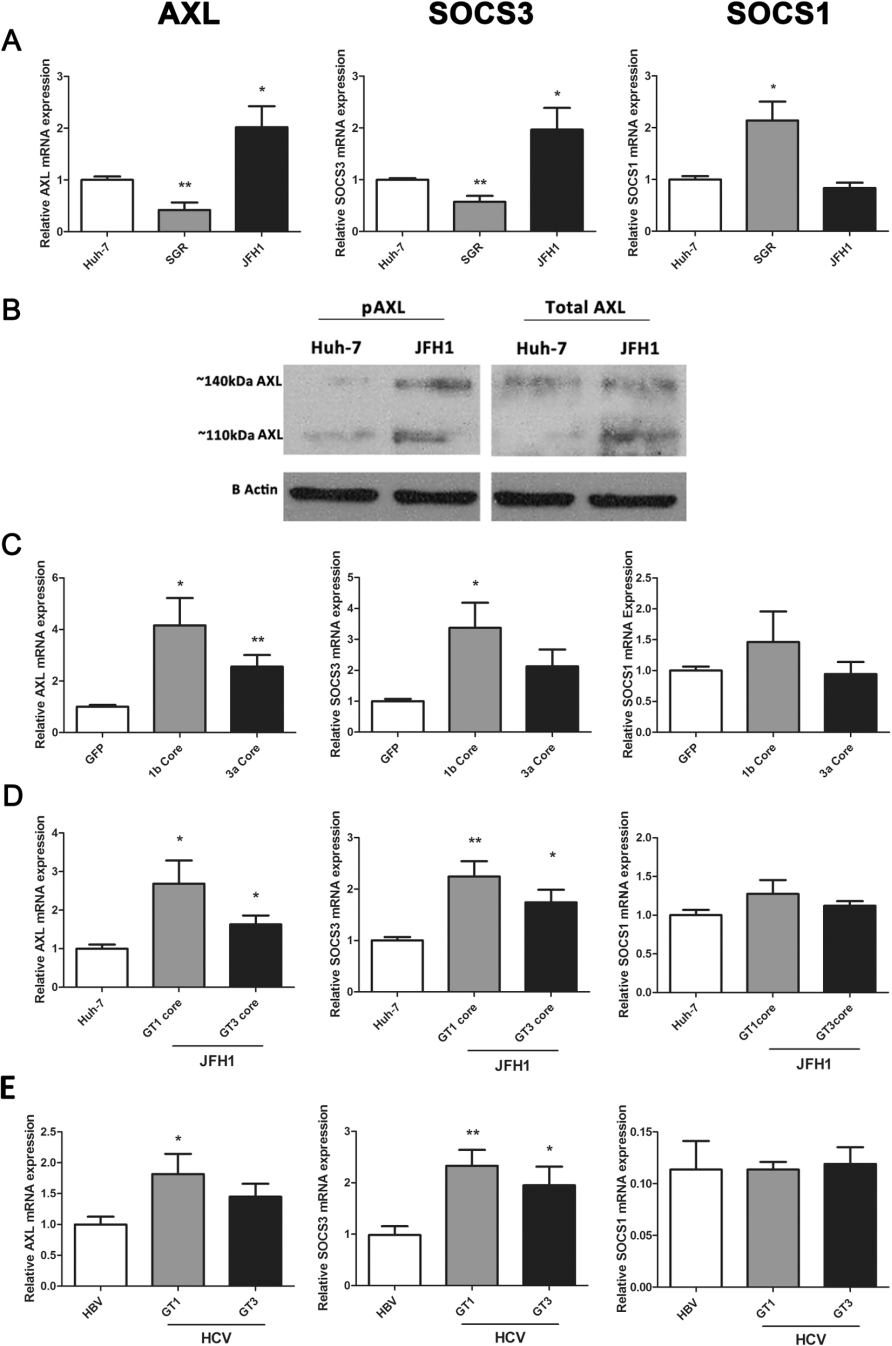
HCV infection mediates AXL expression *in vitro* and *in vivo*. *AXL* and *SOCS3* expression are significantly increased in JFH1 infected Huh-7 cells, but decreased in cells containing a genotype 2a HCV subgenomic replicon (SGR), which lacks structural proteins. This was demonstrated at the mRNA level by qPCR (A), and confirmed at the protein level by western blot (B). Genotype dependent (GT1>3) induction of *AXL* and *SOCS3* was observed in HepG2 cells transfected with baculovirus expressing HCV core protein (C), as well as in Huh-7 cells infected with core chimeric HCV viruses (D) (* p<0.05, ** p<0.01). Data represents the mean and standard error of three biological replicates, each with duplicate samples.

We previously demonstrated that infection of Huh-7 cells with HCV (JFH1 strain) induces the expression of *AXL* as well as its downstream target *SOCS3* [13]. To further confirm the mechanism by which AXL and SOCS3 are induced during HCV infection, we examined their expression in Huh-7 cells containing a HCV subgenomic replicon (SGR), which expresses only non-structural viral proteins, and in cells containing fully replicating JFH1 virus. Both *AXL* and *SOCS3* were up-regulated approximately 2 fold in JFH1 infected cells (minimum 2 passages, 1 week post infection, 90% infectivity), but were surprisingly down-regulated in a stable cell line of Huh-7s harbouring a genotype 2a SGR [19] (Fig 1A). *SOCS1* demonstrated an opposite expression pattern, being up-regulated 2 fold in cells harbouring the SGR (p<0.05). To determine whether the AXL protein is activated and thus able to induce SOCS3 in JFH1 infected Huh-7 cells, we examined AXL phosphorylation at tyrosine 779 by western blot. Interestingly, both total AXL and phosphorylated AXL were up-regulated in JFH1 infected cells (Fig 1B). Furthermore, both glycosylation states of AXL, represented by bands at approximately 110 and 140 kDa, were increased in JFH1 infected cells. These data suggest that AXL may drive SOCS3 expression in these cells, as has been shown in other models [12,20].

Because the HCV structural protein core has been shown to induce SOCS3 expression *in vitro* [6], we examined AXL/SOCS3 expression in HepG2 cells over-expressing genotype 1b and 3a HCV core protein, using a baculovirus expression system. At 48 h post-electroporation, genotype 1b core significantly up-regulated both *AXL* and *SOCS3* by 4 and 3 fold respectively, while genotype 3a core up-regulated *SOCS3* alone by 2 fold (Fig 1C). Next, we examined *AXL* and *SOCS3* expression in Huh-7 cells harbouring chimeric HCV constructs, which express genotype 1b or 3a core in a JFH1 (genotype 2a) virus, and observed similar results. While both chimeric viruses replicated at lower levels than wild type JFH1 (S1 Fig), both induced significant *AXL* and *SOCS3* up-regulation, albeit more potently following infection with the genotype 1b chimera (Fig 1D). Neither core expression nor chimeric virus induced the expression of *SOCS1*.

### HCV induced AXL expression is mediated by antiviral and inflammatory signalling pathways

**Fig 2:**
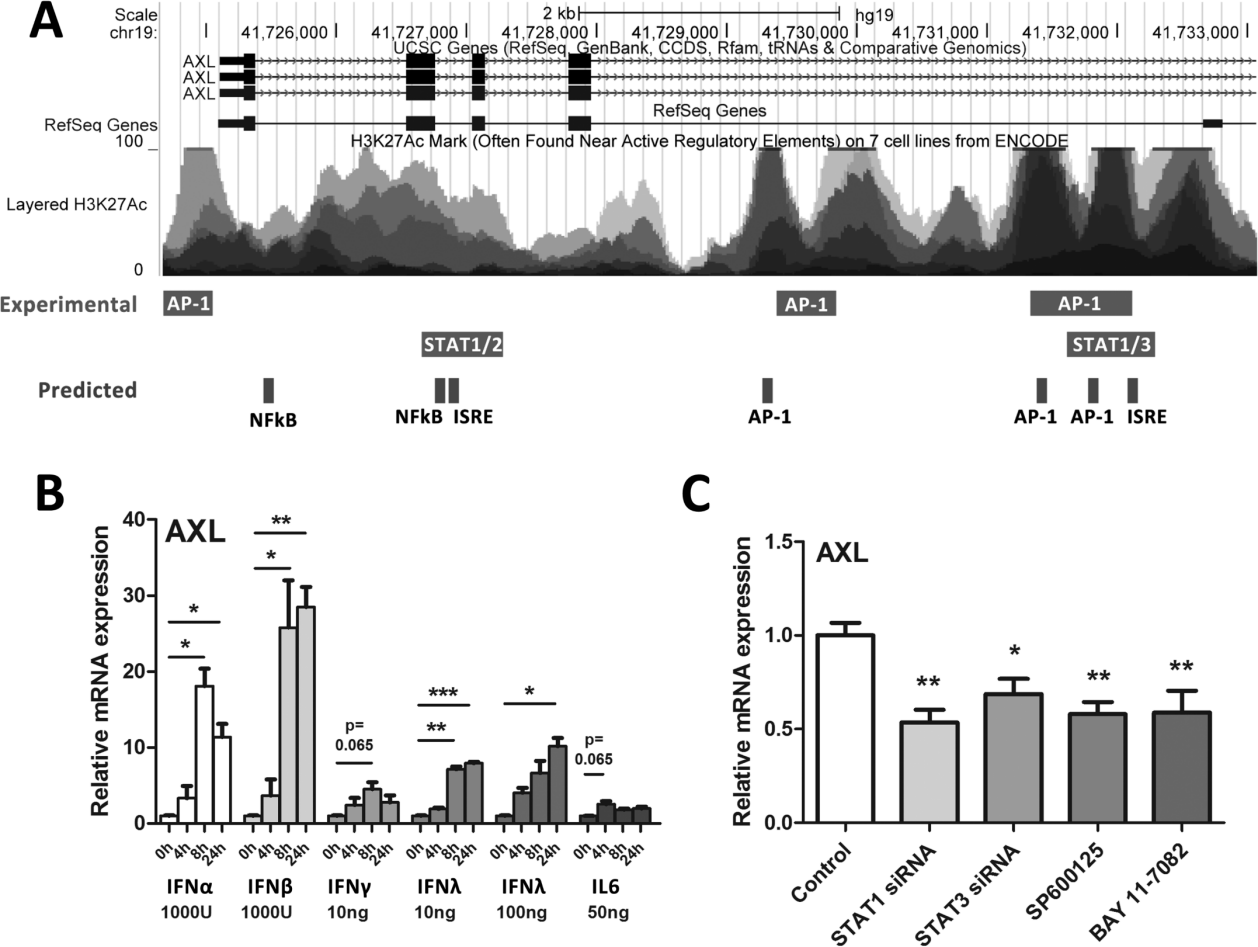
AXL expression is mediated by multiple innate immune signalling pathways. To identify inflammatory transcription factor binding sites within the AXL promoter/enhancer region that may mediate AXL expression, the UCSC genome browser was interrogated for experimental ChIP binding sites (A). Furthermore, the ECR browser and rVista were used to identify evolutionarily conserved regions and to predict transcription factor binding sites, respectively. Huh-7 cells were treated with a range of antiviral cytokines for 24 h then AXL expression measured by qPCR (B). IFNs α, β, and λ up-regulated AXL more potently than pro-inflammatory cytokines IFNγ and IL6 (average of two biological replicates in duplicate for each). (C) To inhibit potential signalling components that induce *AXL*, Huh-7 cells were treated with 10nM siRNA against STAT1/STAT3 or chemical inhibitors of JNK (SP600125) or NFκB (BAY11-7082) for 24 h. Inhibition of STAT1, STAT3, JNK (SP600125) and NFκB (BAY 11–7082) all significantly reduced HCV induced AXL expression (* p<0.05, ** p<0.01, *** p<0.001) (average of three biological replicates in duplicate). Data represents the mean and standard error.

To determine cell factors that may influence AXL expression, the UCSC genome browser was used to identify experimentally validated transcription factor binding sites within the *AXL* promoter/enhancer region (S2 Fig) [16]. The ECR Browser was used to identify evolutionarily conserved regions within the promoter and introns of the *AXL* gene, and rVISTA was used to find conserved transcription factor binding sites, using the *Rattus Norvegicus* genome as a reference (S3 Fig) [17,18]. A number of transcription factors that mediate innate immune signalling, including STAT1, 2 and 3, as well as AP-1 signalling components (c-Fos, c-Jun), have been shown to bind to the *AXL* gene, principally at intronic sequences in the 5’ region (Fig 2A). Furthermore, a number of evolutionarily conserved transcription factor binding sites were predicted in overlapping regions, including additional NFκB binding sites.

To identify which innate immune cytokines that are typically induced by HCV can up-regulate AXL expression, we treated Huh-7 cells with IFNs α (1000 U/ml), β (1000 U/ml), γ (10ng/ml) and λ3 (10 and 100 ng/ml) as well as IL6 (50 ng/ml) for 24 h and measured *AXL*. Interestingly, all cytokines tested induced *AXL* expression, albeit to differing degrees, with type I interferons α and β having the strongest effect (Fig 2B). Potential IFNα mediated induction of other members of the TAM family of receptor tyrosine kinases (Tyro3 and Mer) was investigated, but only *AXL* was found to be an ISG (S4 Fig).

To better understand how AXL is induced following HCV infection, we inhibited multiple innate immune and inflammatory signalling pathways in JFH1 infected Huh-7 cells, using either siRNA or chemical inhibitors. STAT1 and STAT3 siRNAs were used to inhibit IFN and IL6 induced signalling pathways; SP600125 was used to inhibit JNK and downstream AP-1 signalling and BAY11-7082 was used to inhibit NFκB signalling. The efficacy of siRNA gene knockdown and all chemical inhibitors was confirmed by STAT1/3 western blot and NFκB/AP-1 promoter activation using luciferase reporter plasmids respectively (S5 Fig). Inhibition of STAT1 and 3, JNK or NFκB signalling each reduced *AXL* expression by approximately 2 fold (Fig 2C), suggesting that HCV mediated AXL expression is complex and is likely mediated by multiple transcription factors. Expression of SOCS1 and SOCS3 was unaffected (data not shown).

### AXL knockdown reduces SOCS3 expression but does not affect JFH1 replication

**Fig 3:**
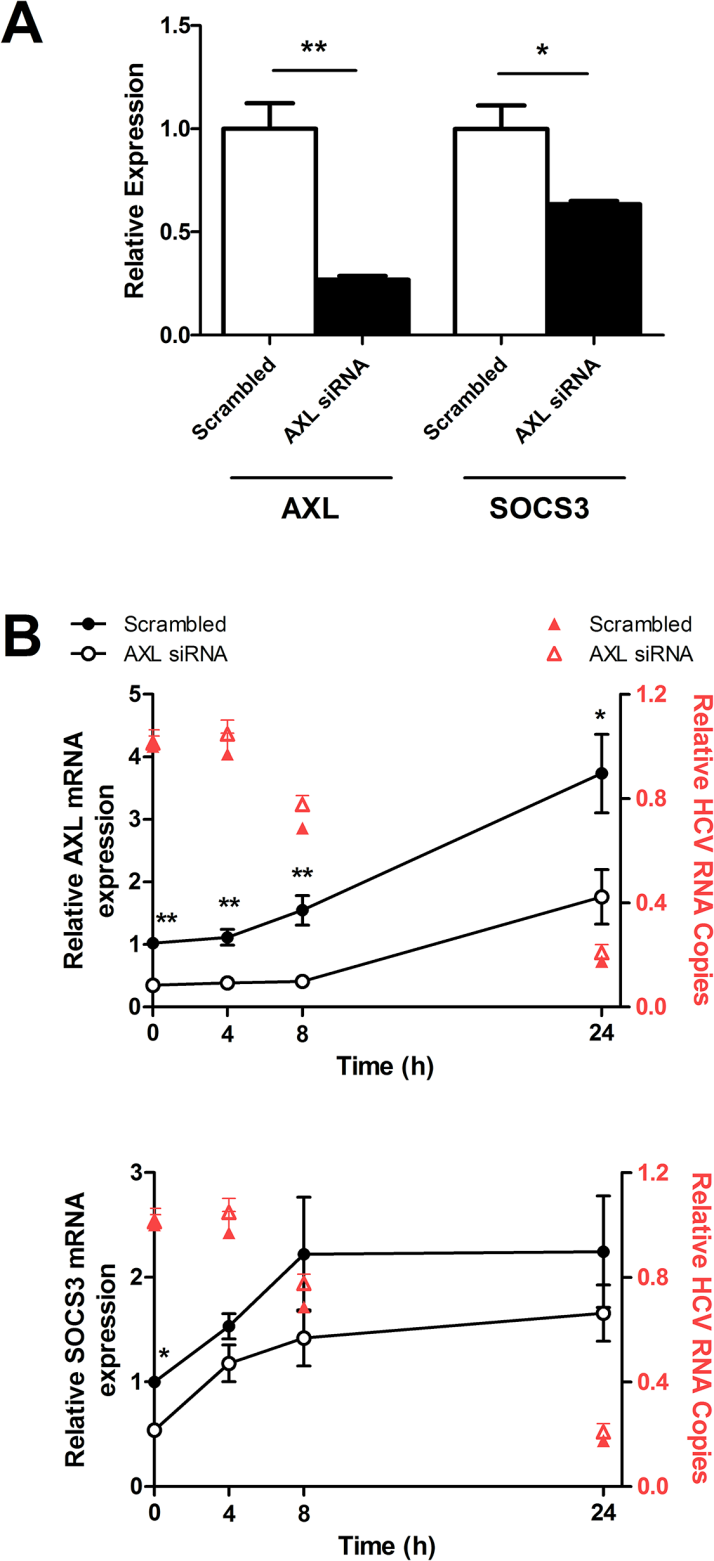
AXL knockdown reduces SOCS3 expression but does not affect HCV replication. *AXL* specific siRNA knockdown reduced *AXL* expression by approximately 75%, also resulting in a significant reduction of *SOCS3* by approximately 40% (A). *AXL* knockdown was maintained for 24 h after 50 U/ml IFNα stimulation, with a non-significant trend towards reduced *SOCS3* expression (B). *AXL* knockdown did not reduce HCV viral replication following IFNα treatment (red triangles) (* p<0.05, ** p<0.01). Data represents the mean and standard error of two biological replicates performed in duplicate.

To determine the role of AXL in SOCS3 induction, as well as in the interferon response to HCV infection, *AXL* was knocked down in JFH1 infected Huh-7 cells using 10nM AXL siRNA. SiRNA mediated knock down of *AXL* by approximately 75% significantly reduced *SOCS3* expression, by approximately 40% (Fig 3A). *AXL* knockdown was maintained for 24 h after 50 U/ml IFNα treatment, while *SOCS3* expression remained down-regulated, albeit modestly (Fig 3B). *AXL* knockdown had no effect on HCV replication, either at baseline or following IFNα treatment (red triangles), nor did it have any effect on ISG expression (data not shown).

### AXL overexpression dampens the ISG response to IFNα in Huh-7 cells

**Fig 4:**
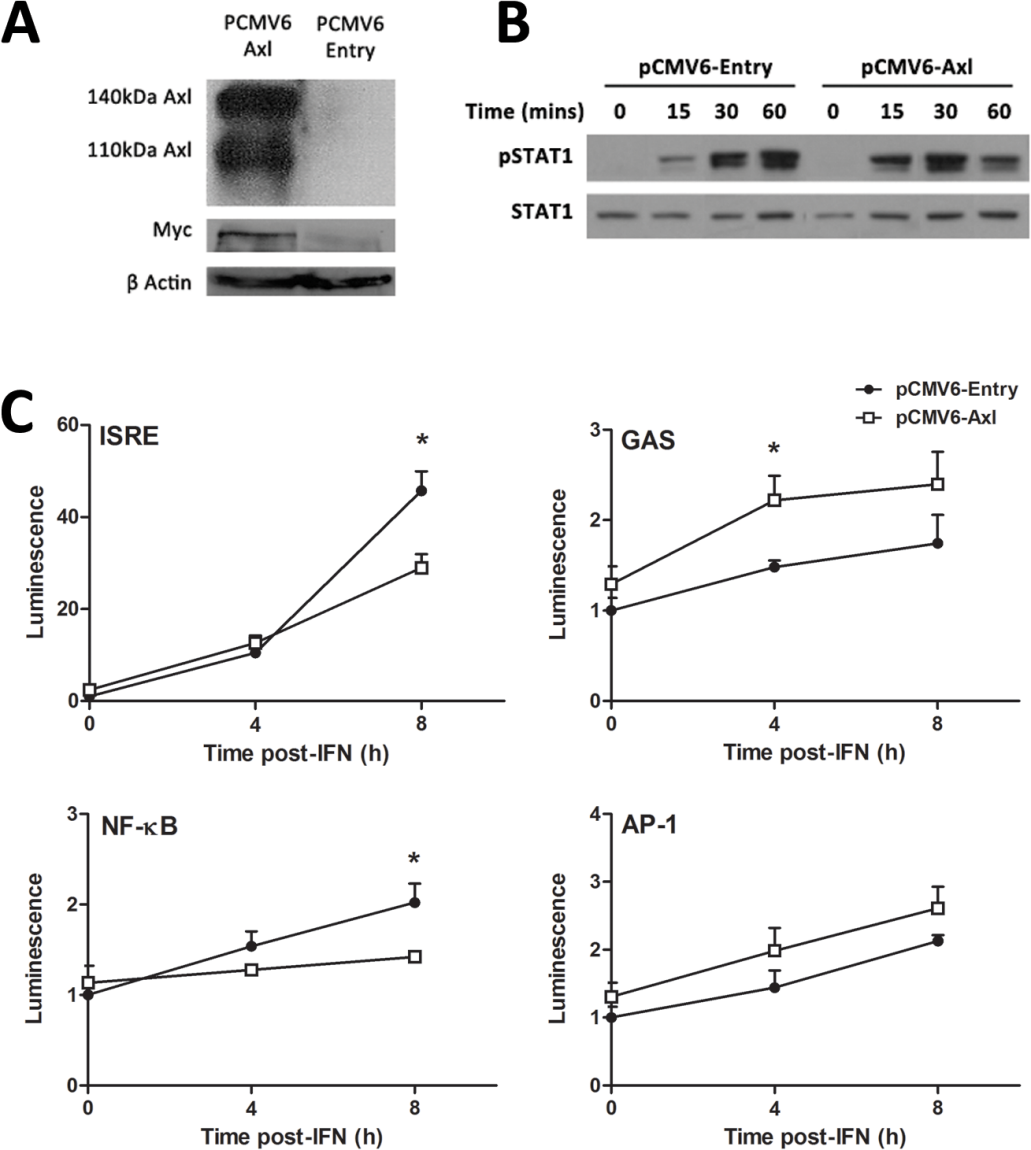
AXL overexpression dampens the response to IFNα. Stable transfection of Huh-7 cells with PCMV6-AXL was confirmed by western blot, using antibodies against AXL or the fusion Myc tag (A). Huh-7 cells overexpressing AXL induced a stronger but more transient phosphorylation of STAT1 (B), with increased phosphorylation at 15 and 30 minutes post-IFN (50 u/ml), but a strong decrease at 1 h (2 replicates). ISRE and NFκB promoter activation was decreased almost 2 fold at 8 h post-IFN treatment in cells overexpressing AXL, while GAS activation were increased 2 fold at 4 h post-IFN treatment (C). No effect on AP-1 promoter activation was observed (* p<0.05). Data represents the mean and standard error of two biological replicates performed in duplicate.

Next, AXL was over-expressed in Huh-7 cells, to determine whether it can influence STAT1 phosphorylation, promoter activation and ISG expression in response to IFNα. Strong AXL overexpression was achieved following stable transfection with the PCMV6-AXL vector, with PCMV6-Entry as a control. This was confirmed by western blot, using antibodies against either AXL or the Myc protein tag (Fig 4A). In cells overexpressing AXL, IFNα (50 U/ml) induced a more transient activation of STAT1, characterised by stronger STAT1 phosphorylation 15 and 30 minutes post treatment, with reduced STAT1 phosphorylation at 60 minutes (Fig 4B). Furthermore, in AXL over-expressing Huh-7 cells, 50 U/ml IFNα induced activation of the interferon stimulated response element (ISRE) and NFκB was significantly blunted 8 h post-treatment, using luciferase reporter constructs (Fig 4C). In contrast, the gamma activated sequence (GAS) activation increased almost two fold at 4 h post treatment and no change in activation of the AP-1 promoter was observed in AXL overexpressing cells. Together, these data agree with previous reports in dendritic cells, suggesting that AXL highjacks signalling from the type I IFN receptor, thereby limiting ISRE and NFκB activation, and also produces STAT1 homodimers that bind the GAS sequence [12].

**Fig 5:**
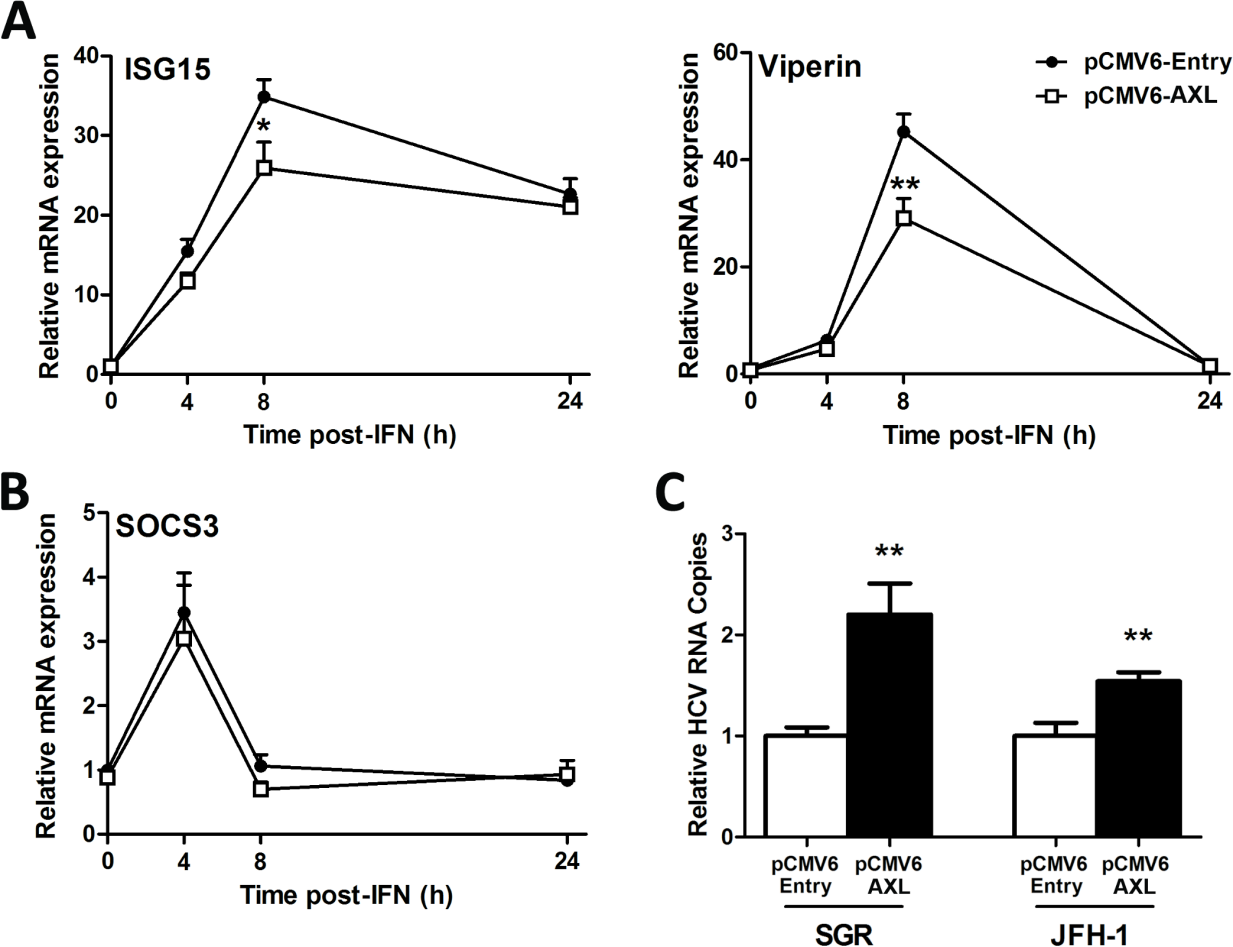
AXL overexpression dampens the antiviral response. Following treatment with 50 U/ml IFNα, Huh-7 cells stably expressing AXL demonstrated blunted ISG15 and viperin expression (A), but no change in SOCS3 (B), compared to control cells. Stable AXL overexpression also mediated an increase in full length JFH1 and SGR viral replication at 24 h post-transfection (C) (* p<0.05, ** p<0.01). Data represents the mean and standard error of three biological replicates performed in duplicate.

We next examined ISG expression in response to IFNα to determine whether modulation of promoter activity by AXL (Fig 4) also reduces ISG expression. Huh-7 cells over-expressing AXL were significantly less responsive to 50 U/ml IFNα, with reduced induction of *ISG15* and *viperin* (Fig 5A). Surprisingly, AXL overexpression had no effect on expression of *SOCS1* (data not shown) or *SOCS3*(Fig 5B), suggesting that in this model, AXL alone is likely responsible for regulation of IFNα signalling. To determine whether AXL exerts IFN regulatory and thus pro-viral effects, both control and AXL over-expressing Huh-7 cell lines were transfected with RNA for either full length HCV virus (JFH1 strain) or a JFH1-derived SGR, lacking structural. AXL over-expression increased viral replication over 2 fold for the SGR and 1.5 fold for JFH1 (Fig 5C), suggesting that AXL can weaken the antiviral response to HCV *in vitro*.

### AXL and SOCS3 are up-regulated in HCV infected liver

**Fig 6:**
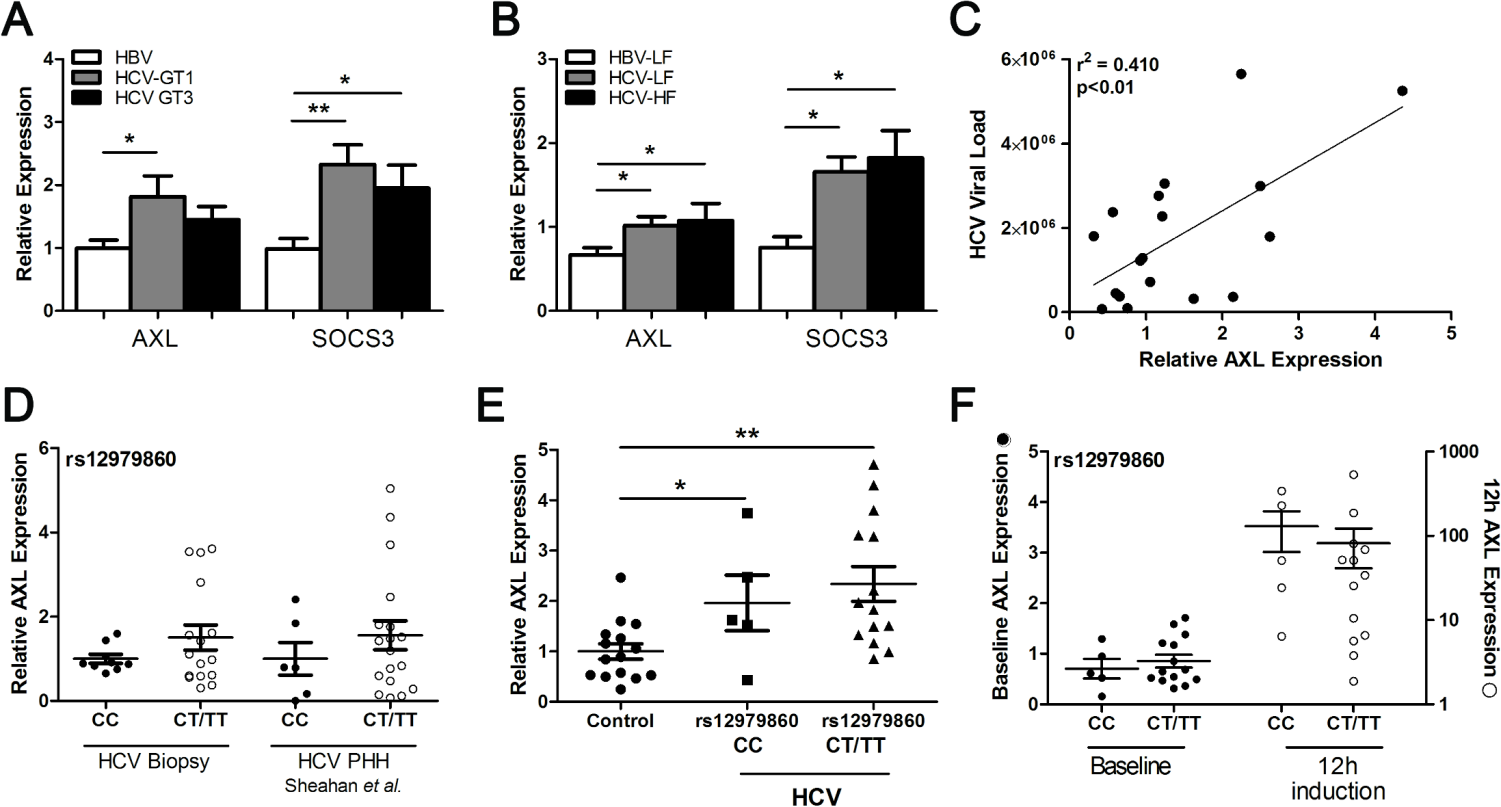
HCV genotype and IFNL3 SNP modulate AXL expression. *AXL* and *SOCS3* expression were examined in liver biopsies from patients with chronic HBV or chronic HCV infection, to look for virus specific differences in gene induction. HCV Genotype 1 infected livers demonstrated significantly higher expression of both *SOCS3* and *AXL* (A), with no effect of liver fibrosis grade on expression of either *SOCS3* of *AXL* in HCV infected livers (B). *AXL* expression also demonstrated a strong correlation with viral load, supporting the direct role of HCV in AXL induction (C). The rs12979860 *IFNL3* CC SNP correlated with lower baseline hepatic *AXL* expression in genotype 1 HCV infected livers and JFH1 infected PHHs (D). PBMC expression of AXL was up-regulated in HCV infected individuals, and similar to hepatic expression, was lower in patients possessing the CC genotype (E). Stronger *AXL* up-regulation in PBMCs 12 h after the first IFN injection was also observed (F) (* p<0.05, ** p<0.01).

To determine whether our *in vitro* results mimic *in vivo* gene expression, we compared liver biopsies from patients infected with HCV genotype 1 or 3, with HBV infected livers as controls. Both *AXL* and *SOCS3* were significantly up-regulated in genotype 1 infected livers compared to HBV controls, whereas *SOCS3* was only significantly higher in the genotype 3 infected livers (Fig 6A). Both *AXL* and *SOCS3* were significantly increased in HCV infected livers with low (F0-2) or high (F3-4) levels of fibrosis, compared to low fibrosis HBV liver biopsies, indicating that *AXL* expression is not affected by fibrosis stage (Fig 6B). A strong correlation was also observed between hepatic AXL expression and HCV viral load (Fig 6C), suggesting that increased viral replication and subsequent activation of immune signalling pathways may drive AXL expression in the liver.

We next examined *AXL* expression in liver and blood from HCV infected patients, stratified by the IFNL3 rs12979860 SNP. Hepatic *AXL* expression was higher in patients with the non-responder rs12979860 CT/TT genotype (Fig 6D), consistent with published data showing elevated hepatic ISGs in patients possessing the IFNL3 non-responder haplotype [3,14]. To examine the effect of IFNL3 genotype on AXL in a PHH model, we utilized publicly available microarray data examining expression of primary human hepatocytes (PHHs) infected with HCV (Geo Dataset GSE54648). Laser capture microdissection was utilized to compare infected and infection adjacent cells, and was further stratified by IFNL3 rs12979860 genotype [21]. By analysing AXL expression in both infected and adjacent PHHs (gene expression results combined to simulate *in vivo* infection) we found similar results as *in vivo* HCV infection, with elevated *AXL* expression in HCV infected PHH isolated from patients with the “non-responder” rs12979860 CT/TT genotype (Fig 6D).

To determine whether AXL induction is mediated directly by viral infection or by subsequent cytokine expression, we examined AXL expression in infected, adjacent and mock infected PHHs from the same dataset. AXL expression increased in infected cells from day 1 to day 7 (S6 Fig), and was consistently higher in infected cells than either adjacent cells or mock infected controls (p<0.05 vs mock, day 3). This suggests that although cytokines may contribute to AXL expression *in vivo*, AXL up-regulation occurs primarily in infected cells.

*AXL* expression was also significantly elevated in peripheral blood mononuclear cells (PBMCs) from HCV infected individuals (n = 20) compared to healthy controls (n = 15) (p<0.05) (Fig 6E). Furthermore, patients possessing the responder rs12979860 CC genotype displayed lower *AXL* expression than CT/TT carriers, in agreement with hepatic *AXL* expression. Finally, induction of *AXL* 12 h after the first dose of pegylated IFN was stronger in PBMCs from patients possessing the responder CC genotype, compared with CT/TT non-responder patients (Fig 6F).

## Discussion

We previously demonstrated that AXL is up-regulated following HCV infection *in vitro*, and may contribute to SOCS3 expression and interferon refractoriness [13]. We now demonstrate that AXL is induced in a genotype dependent manner (GT1>3) *in vitro* as well as *in vivo*, and is likely induced by IFNs and other inflammatory mediators. AXL knockdown showed little effect on IFN signalling, however AXL overexpression reduced Huh-7 responsiveness to IFN, as well as the antiviral response against HCV.

Our current findings build on our previous report that HCV induces AXL expression *in vitro*. We have now clarified (1) that viral replication alone, in the absence of HCV structural proteins, is not sufficient to induce AXL/SOCS3 expression, and (2) that genotype 1 core protein, either alone or as part of a chimeric virus, is a more potent inducer of AXL/SOCS3 than genotype 3 core (Fig 1). The similar expression pattern of AXL and SOCS3 suggests that AXL may induce SOCS3 in our model, particularly in the presence of genotype 1 core protein, and we have confirmed the association with HCV genotype in human liver tissue (Fig 6). This suggests that AXL may drive increased expression of SOCS3, which has been implicated in genotype 1 mediated insulin resistance and treatment non-response [22–25].

Because type I IFN has been shown to induce AXL in myeloid cells in the blood [11,12,26], we sought to determine whether we could reproduce AXL induction in Huh-7 cells. Surprisingly, the inflammatory cytokines IL6 and IFNγ only modestly up-regulated *AXL*, while type I/III IFNs (β>α>λ) potently induced its expression. Knockdown of STAT1/3 or chemical inhibition of JNK or NFκB signalling all reduced *AXL* expression in JFH1 infected Huh-7 cells, suggesting that AXL induction is mediated by multiple transcription factors, similar to the IFNβ enhanceosome [27]. Because HCV induces oxidative stress, inflammation, and a strong type III immune response in the liver, it is not surprising that the activation of these transcription factors following infection *in vitro* induced *AXL* expression [28–33].

Our experiments modulating AXL expression yielded conflicting results, concerning effects on ISGs and SOCS3. Although AXL knockdown reduced baseline SOCS3 expression, AXL overexpression had no effect on SOCS3. Conversely, ISG expression was not affected by AXL knockdown but was reduced following AXL overexpression. Together, these data suggest that AXL induced SOCS3 expression does not affect ISG expression, but rather AXL itself interferes with IFN signalling in Huh-7 cells. AXL knockdown did not increase ISG expression, suggesting that baseline expression of AXL in Huh-7 cells is insufficient to affect IFN signalling. Nonetheless, AXL overexpression significantly decreased IFN induced ISG expression and increased viral replication in Huh-7 cells, indicating that it has the capacity to interfere with signal transduction in hepatocytes. As such, we propose a model where in hepatocytes AXL binds to IFNAR1 to inhibit STAT1:2 heterodimer formation (reducing ISRE activation, ISG expression), induces STAT1 homodimer formation (increasing GAS activation), as demonstrated by Rothlin *et al*. [12] in dendritic cells, but does *not* induce SOCS1/SOCS3 expression.

Finally, we observed a trend towards lower hepatic *AXL* expression and a stronger ISG response in blood PBMCs among patients with the favourable rs12979860 IFNL3 allele (CC), although this was not statistically significant, possibly due to insufficient statistical power. These data mirror previous reports, that the *IFNL3* non-responder haplotype is associated with higher baseline ISGs in the liver and a weaker immune response in the blood [14,34,35], but provide a novel mechanism to explain this. Because AXL expression is induced by type I and III IFNs, it is likely that the recently described IFNL4 also induces AXL. IFNL4 is only expressed in the livers of patients with the unfavourable SNP (ss469415590), so IFNL4 driven AXL could potentially reduce the antiviral response to exogenous IFN in these individuals. Nonetheless, because AXL is an ISG, it may have the ability to regulate its own expression, and possibly the expression of other negative regulators such as USP18. This adds another layer of complexity to IFN regulation, and clearly requires further study.

The consequences of HCV mediated AXL up-regulation are not limited to regulation of IFN signalling. Up-regulation of AXL is well documented in hepatocellular carcinoma (HCC), and contributes to HCC cell line proliferation, migration and invasion [36–38], through activation of AKT and MAPK signalling pathways (reviewed in [39]). Moreover, AXL serves as an entry factor for a number of enveloped viruses, including the related dengue flavivirus [40–42]. If the same is true for HCV, immune-driven AXL expression in hepatocytes in the HCV infected liver could facilitate the spread of the virus into uninfected cells.

In summary, we have shown that AXL is induced by HCV infection, and is capable of regulating the ISG response to HCV in hepatocytes. The immunomodulatory role of AXL in the context of IFNL3/4 polymorphisms remains uncertain, but may provide a mechanism for the differences observed in hepatic and blood ISG regulation, based on the dominant IFNs in each cell type.

## Supporting Information

S1 Fig Relative HCV RNA replication in wild type JFH1 and genotype 1 and 3 core chimera infected Huh-7 cells. Intracellular HCV RNA content in JFH1 core chimeras was approximately 25% of wild type JFH1 virus. Reduced replication capacity is likely the result of decreased viral fitness from intergenotypic core replacement.
(TIF)

S2 Fig Transcription factor binding to the AXL promoter and enhancer. Experimental ChIP studies have demonstrated a large degree of transcription factor binding within the 4^th^ intron of AXL, suggesting the presence of an enhancer region.
(TIF)

S3 Fig Conserved AXL sequence in *Homo sapiens* and *Rattus norvegicus*. The 5’ portion of the AXL gene is conserved in rats but not in mice as demonstrated by the ECR browser.
(TIF)

S4 Fig AXL is the only TAM receptor that is an ISG. Expression of AXL, but not TYRO3 or MER, was significantly up-regulated in Huh-7 cells 8 h after treatment with 100 U/ml IFNα, (*** p<0.001).
(TIF)

S5 Fig Confirmation of siRNA and chemical inhibition of inflammatory pathways. Western blot showing that both STAT1 (A) and STAT3 (B) expression were reduced by at least 50% following siRNA treatment. Promoter activation of AP-1 (C) and NFκB (D) luciferase reporters were drastically reduced using 50 μM SP600125 and 25 μM BAY11-7082 respectively.
(TIF)

S6 Fig AXL expression in HCV infected primary human hepatocytes. AXL expression is up-regulated following HCV infection of primary human hepatocytes, compared to adjacent uninfected cells and mock infected cells (p<0.05, infected vs mock, day 3). Data taken from Geo Dataset GSE54648 using hepatocytes isolated by laser capture microdissection.
(TIF)
