# Supplementary figures and images for: Hepatitis C Virus Driven AXL Expression Suppresses the Hepatic Type I Interferon Response
Source: PLoS One. 2015 Aug 27;10(8):e0136227. doi: 10.1371/journal.pone.0136227 (PMC4551482; doi:10.1371/journal.pone.0136227)

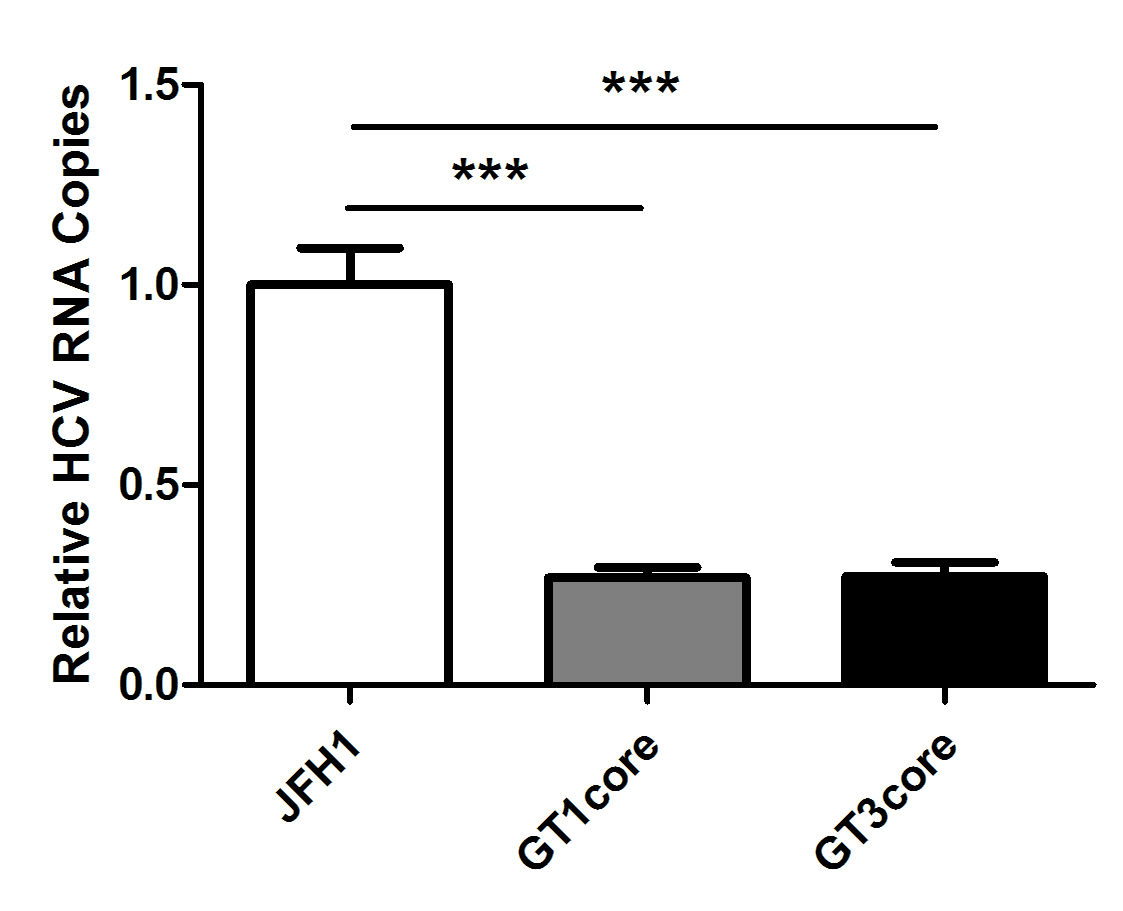

Supplement: S1 Fig — Intracellular HCV RNA content in JFH1 core chimeras was approximately 25% of wild type JFH1 virus. Reduced replication capacity is likely the result of decreased viral fitness from intergenotypic core replacement. (TIF) [file pone.0136227.s001.tif]

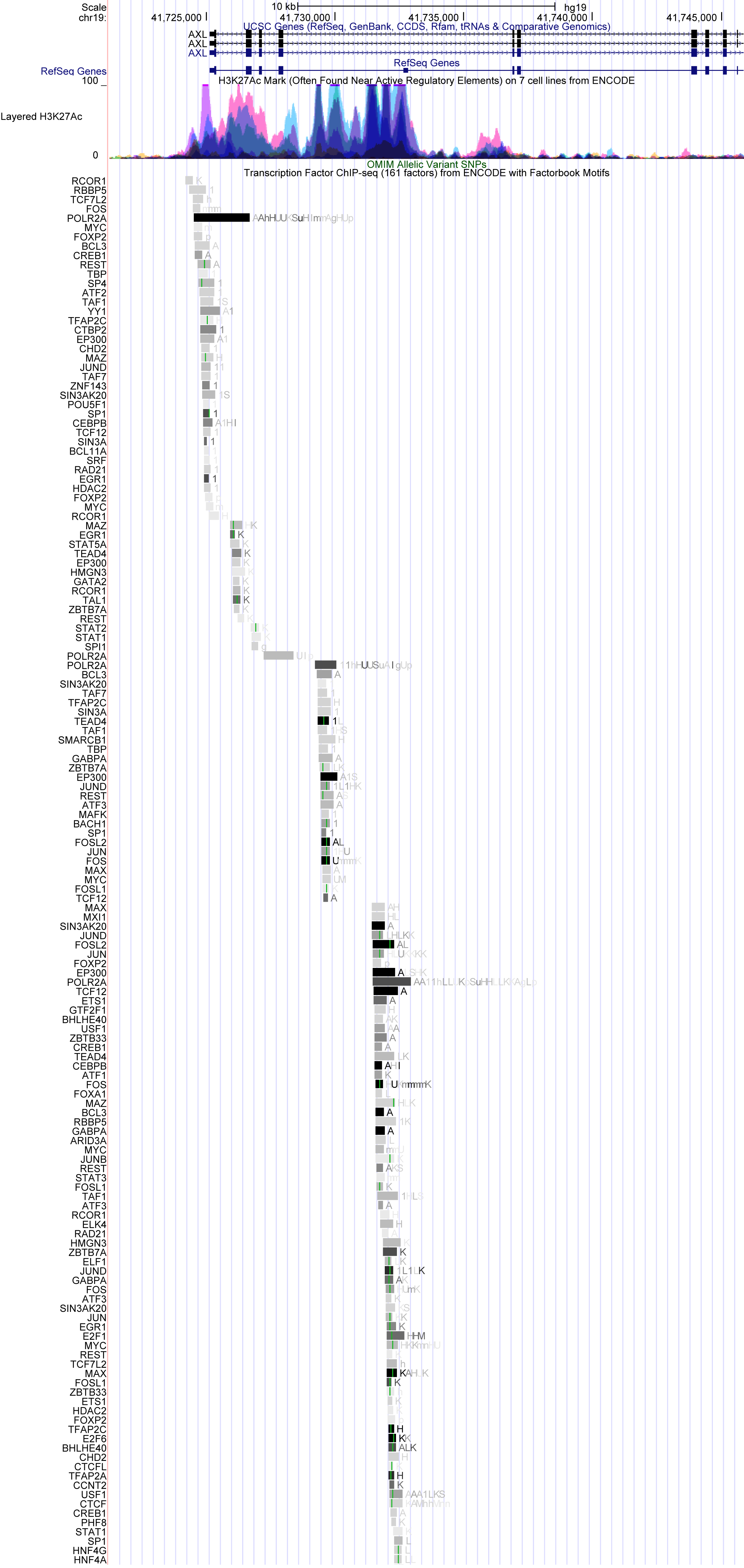

Supplement: S2 Fig — Experimental ChIP studies have demonstrated a large degree of transcription factor binding within the 4th intron of AXL, suggesting the presence of an enhancer region. (TIF) [file pone.0136227.s002.tif]

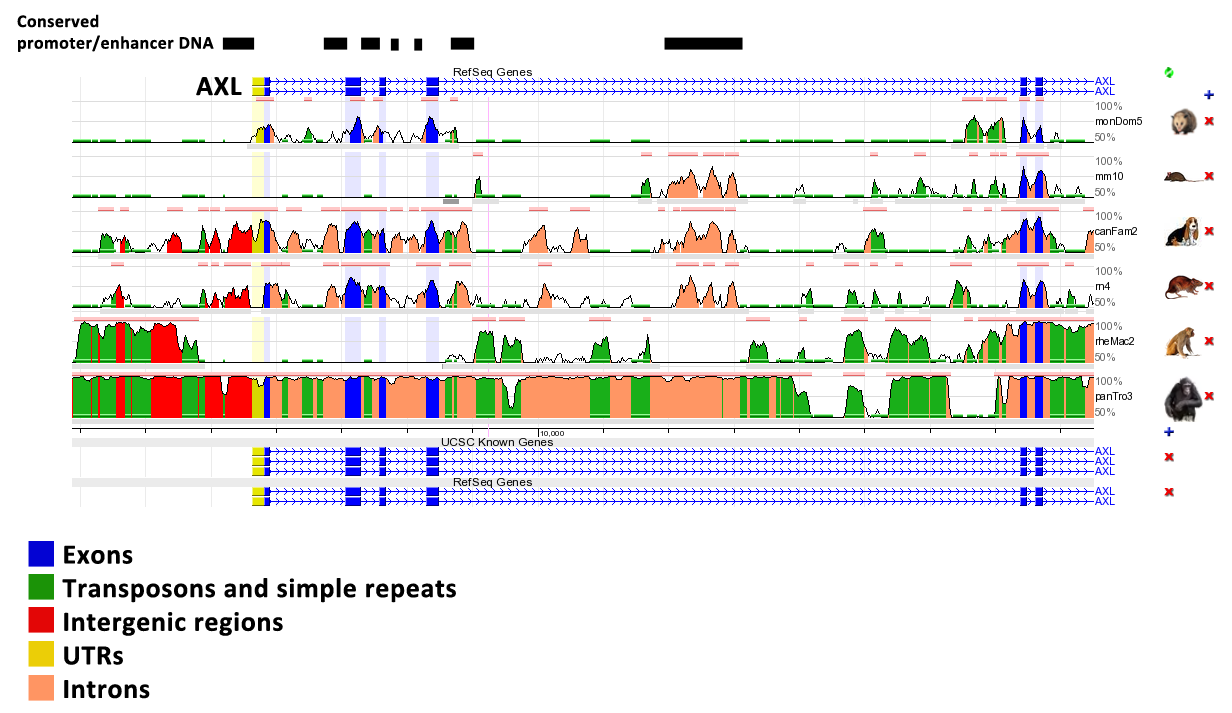

Supplement: S3 Fig — The 5’ portion of the AXL gene is conserved in rats but not in mice as demonstrated by the ECR browser. (TIF) [file pone.0136227.s003.tif]

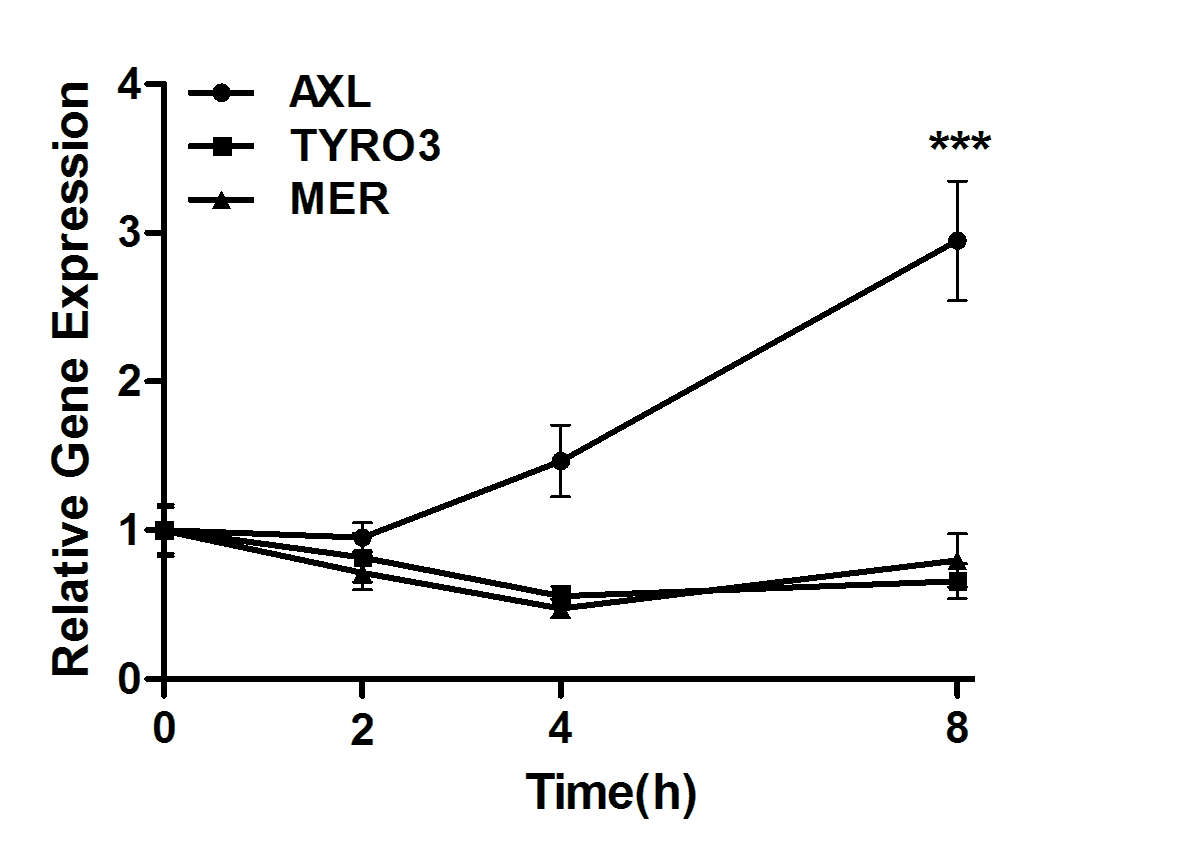

Supplement: S4 Fig — Expression of AXL, but not TYRO3 or MER, was significantly up-regulated in Huh-7 cells 8 h after treatment with 100 U/ml IFNα, (*** p<0.001). (TIF) [file pone.0136227.s004.tif]

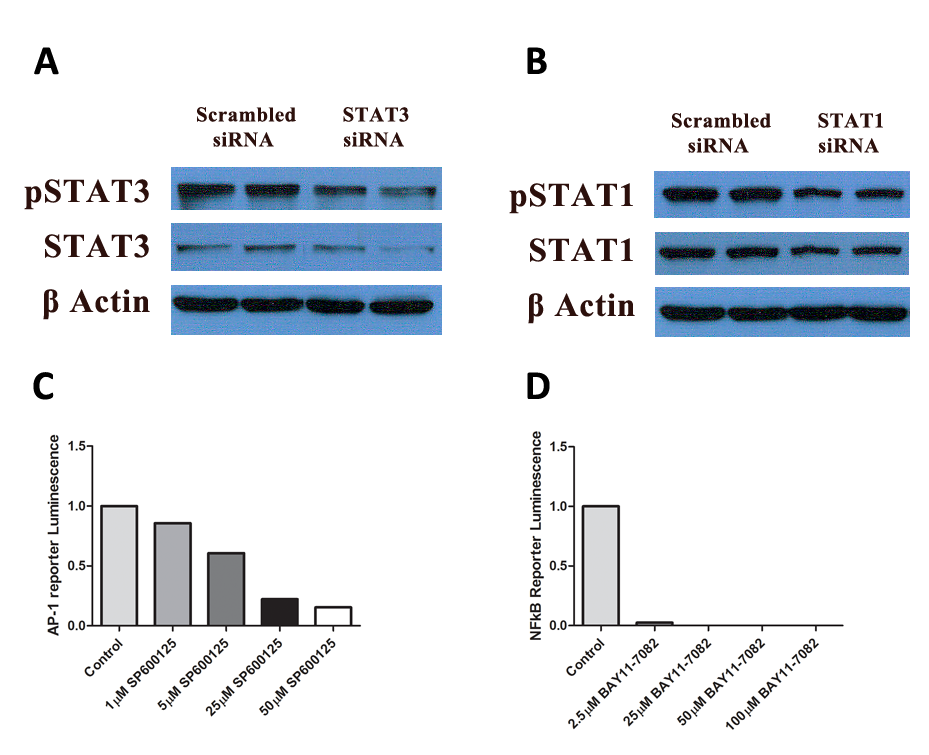

Supplement: S5 Fig — Western blot showing that both STAT1 (A) and STAT3 (B) expression were reduced by at least 50% following siRNA treatment. Promoter activation of AP-1 (C) and NFκB (D) luciferase reporters were drastically reduced using 50 μM SP600125 and 25 μM BAY11-7082 respectively. (TIF) [file pone.0136227.s005.tif]

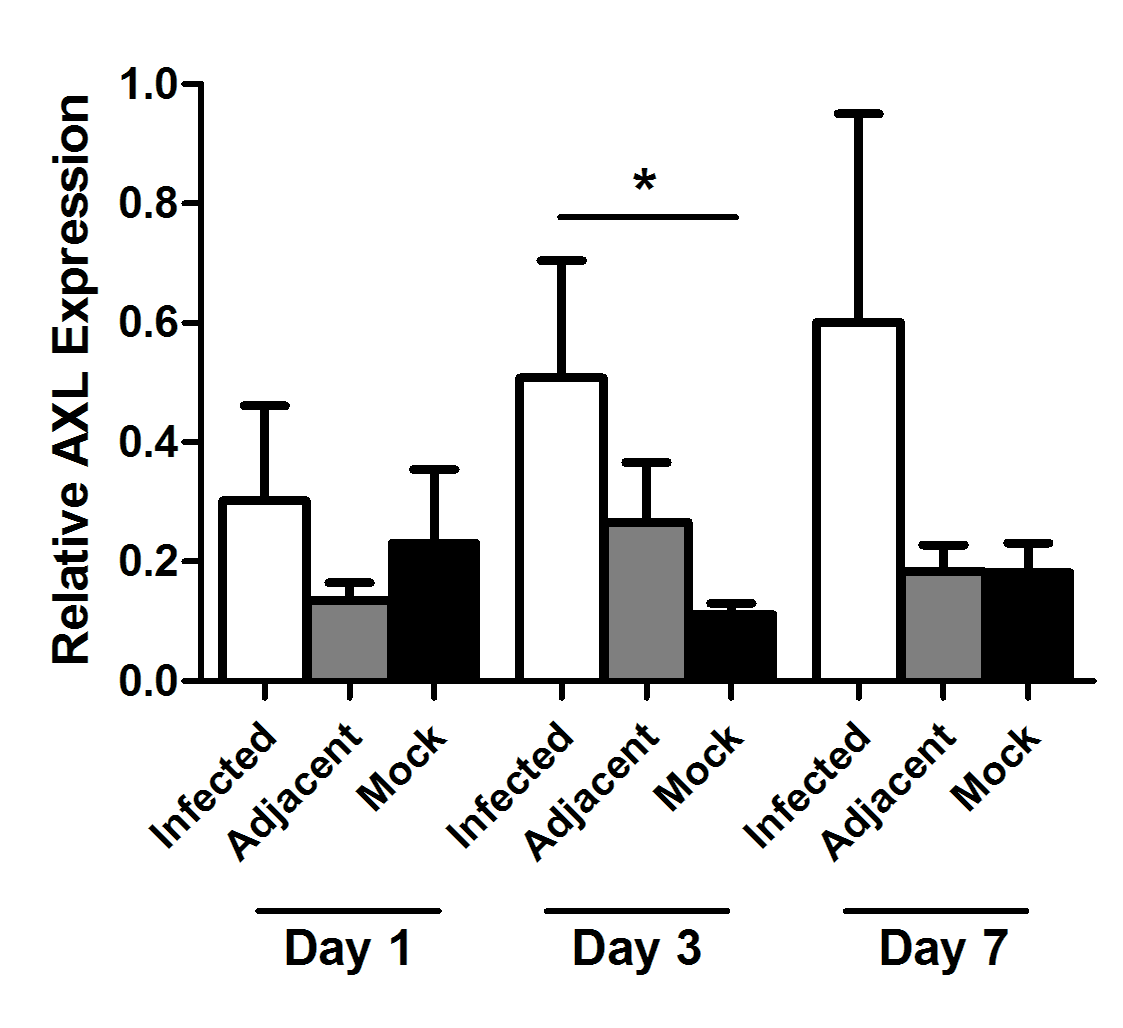

Supplement: S6 Fig — AXL expression is up-regulated following HCV infection of primary human hepatocytes, compared to adjacent uninfected cells and mock infected cells (p<0.05, infected vs mock, day 3). Data taken from Geo Dataset GSE54648 using hepatocytes isolated by laser capture microdissection. (TIF) [file pone.0136227.s006.tif]
